# Analysis of Lactation Performance and Mastitis Incidence in High- and Low-Yielding Dairy Cows Using DHI Data

**DOI:** 10.3390/ani15172495

**Published:** 2025-08-25

**Authors:** Qijun Zhou, Zijian Geng, Shuai Lian, Jianfa Wang, Rui Wu

**Affiliations:** 1 College of Animal Science and Veterinary Medicine, Heilongjiang Bayi Agricultural University, No. 5 Xinfeng Road, Daqing 163319, China; 2 China Key Laboratory of Bovine Disease Control in Northeast China, Ministry of Agriculture and Rural Affairs, Daqing 163319, China; 3 College of Biology and Agriculture, Jiamusi University, Jiamusi 154007, China

**Keywords:** Dairy Herd Improvement, somatic cell count, dairy cow, bovine mastitis

## Abstract

The DHI data is crucial for monitoring the udder health of dairy cows during the breeding process. This study aimed to investigate the factors influencing milk production in dairy cows throughout this period. We analyzed DHI data from Holstein dairy cows in the Heilongjiang region, alongside the incidence of mastitis. The findings revealed that high-yielding cows demonstrated significantly higher peak milk yield days, peak milk yield, urea nitrogen levels, 305-day milk yield, and persistency (*p* < 0.0001) compared to their low-yielding counterparts. Conversely, high-yielding cows exhibited lower protein rates, fat-to-protein ratios, and milk fat rates (*p* < 0.0001). Additionally, the somatic cell count (SCC) in high-yielding cows was significantly lower than that in low-yielding cows (*p* < 0.0001). The multivariate linear regression analysis of the DHI data indicated that parity was the primary determinant affecting both milk yield and SCC. Statistical analysis of cows with clinical mastitis revealed that those experiencing a single episode of clinical mastitis during the lactation period were predominantly in their first and second parities, while recurrent cases were primarily observed in the second and third parities. These results suggest that as the number of lactations increases, the SCC also rises, reflecting the cumulative impact of parity on the udder health of dairy cows.

## Simple Summary

Dairy Herd Improvement (DHI) represents a well-established and comprehensive approach to managing udder health and production activities within dairy cattle herds. By systematically organizing and analyzing DHI data, this method effectively informs strategies for udder health management on dairy farms, optimizes production management processes for dairy cows, and aids in identifying factors associated with both high and low milk-yielding cows, as well as issues impacting udder health. Consequently, this enhances the overall economic viability of dairy operations.

## 1. Introduction

In practical production, dairy farms often employ prevention and control to maintain the udder health of dairy cows. The Dairy Herd Improvement (DHI) system is one of the scientific and technological methods used to achieve this goal [1]. Initially known as DHI, this scientific management system involves the regular testing of lactation performance and milk composition in dairy cows, along with the maintenance of comprehensive records (Appendix A). Specifically, a laboratory milk analyzer is used to evaluate milk components and somatic cell count (SCC). Measurements are taken monthly, and by integrating data on genetic breeding, nutrition, mastitis prevention, and feeding management, a comprehensive assessment of individual cows and the entire herd is conducted. This integrated information enables timely adjustments to feeding strategies, optimizing production potential. Furthermore, it supports standardized, scientific, precise, and intelligent management practices on dairy farms. Notably, DHI programs play a vital role in maintaining udder health, enhancing milk quality, and promoting the sustainable development of the dairy industry [2]. Although DHI data is collected monthly, long-term monitoring is valuable for genetic breeding. While the monthly collection frequency limits its use for real-time monitoring, it can still be linked to viral bovine mastitis in the short term—for example, sudden increases in SCC can serve as early warning signs, allowing for targeted screening when combined with viral testing [3].

The criteria of DHI technology indicate the production performance and udder health of dairy cows. Effectively managing herd udder health through the DHI system plays a crucial role in ensuring dairy product safety at its source [4]. The milk component analyzer can detect the SCC in milk, providing an early warning for cows suffering from mastitis and subclinical mastitis. By promptly isolating affected cows, the spread of disease within the herd can be effectively prevented. This, in turn, enhances the quality of dairy products, ensures their safety, and protects the health rights of consumers [5]. Additionally, when any part of a dairy cow’s body undergoes physiological or pathological changes, it first manifests as a decrease in milk production.

By utilizing the DHI analysis report, one can gain a detailed understanding of the individual milk yield changes for each cow. This provides a direct or indirect means of monitoring and assessing the udder or overall health status of the cows. It helps in promptly identifying sick cows, reduces treatment costs, and effectively decreases the culling rate of cows. As a result, the economic benefits of the dairy farm are improved [6].

## 2. Materials and Methods

### 2.1. Basis and Method of Grouping

To facilitate data organization and subsequent DHI data analysis, grouping was conducted in this study based on seasons, parities, milk yield, lactation stages, and SCC, as detailed below. Seasons: January to December. Parities: 1, 2, 3, 4, and ≥5 parity. Milk Yield: Milk production is grouped according to the average value during the lactation period, High-yielding cows (≥35 kg/day), low-yielding cows (20 kg/day < yield < 30 kg/day). Lactation Stages: 41–100, 101–200, 201–305, and >305 days.

Clinical mastitis data for dairy cows come from records kept by veterinarians stationed on the farm, with the following criteria for diagnosis: SCC Mastitis Criteria: negative (≤2 × 10^5^ cells/mL), weakly positive (2–5 × 10^5^ cells/mL), positive (5 × 10^5^–10^6^ cells/mL), and strongly positive (>5 × 10^6^ cells/mL). In the milking parlor, milk inspectors identify newly occurring cases of mastitis based on whether there are abnormal changes in the milk within the mammary gland and whether the udder shows signs of redness, swelling, heat, pain, or other abnormalities. Subsequently, milk samples from all cows with mastitis are collected according to sampling standards and sent to the laboratory for California Mastitis Test and pathogen detection.

### 2.2. Laboratory Animals and Data Sources

This study was approved and supported by the Animal Welfare and Ethics Committee of Heilongjiang Bayi Agricultural University (Ethics Approval Number: DWKJXY2024057). The experimental animals were sourced from six large-scale dairy farms in the Heilongjiang region. All Holstein cows were housed, milked, and fed three times a day. Milk samples from lactating cows were collected once a month for DHI testing, with no more than 30 days between tests.

In this experiment, we collected 136,295 DHI data entries from 8708 Holstein cows. The clinical mastitis data were obtained from Ranch A, Ranch B, Ranch C, Ranch D, Ranch E, and Ranch F. The specific statistics regarding lactating cow data and the time span covered at each ranch are presented in Table 1.

### 2.3. Dairy Farm Rearing Environment

The Heilongjiang region is characterized by a temperate and cold temperate continental monsoon climate, which includes spring droughts and low temperatures, hot and rainy summers, autumn floods, early frosts, and prolonged cold winters. All dairy cows on the farms are raised using standardized feeding methods. Sand is used as bedding, with each cow having 10 to 12 square meters of activity space and 1.2 to 1.5 square meters of lying area. Ventilation is prioritized in the summer, while insulation is emphasized in the winter. All farms use automated milking equipment.

### 2.4. Data Processing

A total of 136,295 DHI records from 8708 cows across six ranches were collected. Data with missing or blank entries in the DHI reports were excluded as follows:

(1) Records that lacked parity information were excluded;
(2) Records with a milk yield of less than 1 kg were excluded;
(3) Records without an SCC were excluded;
(4) Records the falling outside the normal lactation days were excluded;
(5) Records of cows with fewer than two consecutive DHI tests were excluded;
(6) Records with SCC values exceeding the established Fossomatic SCC measurement range (1–9999 × 10^4^ cells/mL) were also excluded.

### 2.5. Statistical Analysis Description

The collected data were systematically organized, analyzed, and visualized using Microsoft Excel 2021, IBM SPSS Statistics 27, GraphPad Prism 9.5, Adobe Illustrator 2022, and the R (R version 4.5.1) packages lme4, lmerTest, and emmeans.

The differences in lactation performance between high-yield and low-yield dairy cows during the peak lactation period (1–4 months postpartum) were analyzed using a *t*-test.

The differences in lactation performance among high- and low-producing dairy cows across months, parity, and lactation stages were analyzed using a linear mixed-effects model to compare the two groups on various indicators. The model included fixed effects for parity, days in milk (DIM group), month, and group, as well as their interactions, with a random intercept for individual cows (cow ID). The model was specified as follows:

Y = β_0_ + β_1_(parity) + β_2_(DIM group) + β_3_(month) + β_4_(group) + β_5_(parity × group) +  β_6_(DIM group × group) + β_7_(month × group) + (1|cow ID) + ε.

Fixed effects included parity (categorical variable, with >5 combined into a 5+ group), DIM group, month, treatment group, and their interactions; the random effect was the random intercept for individual cows (cow ID). The dependent variables were nine lactation indicators, including milk fat percentage (%), protein percentage (%), and somatic cell count (×10,000/mL). Type III ANOVA was used to assess the significance of each effect. Least squares means (LS-means) were calculated to estimate marginal means, and group comparisons were conducted within each level of parity, lactation stage, and month. Differences between groups and their 95% confidence intervals were also computed.

Factors affecting high and low milk production and somatic cell count in dairy cows were analyzed using multiple linear regression models, with milk yield and somatic cell count designated as dependent variables, respectively. A stepwise selection method was employed to systematically remove non-significant independent variables, thereby developing regression equations that are both representative and optimally fitted.

Error bars in each figure represent the 95% confidence interval; statistical significance was set at α = 0.05, with results indicated as * *p* < 0.05, ** *p* < 0.01, *** *p* < 0.001, and **** *p* < 0.0001.

## 3. Results

### 3.1. Differences in Production Performance Between High-Yield and Low-Yield Dairy Cows During Peak Lactation

**Figure 1:**
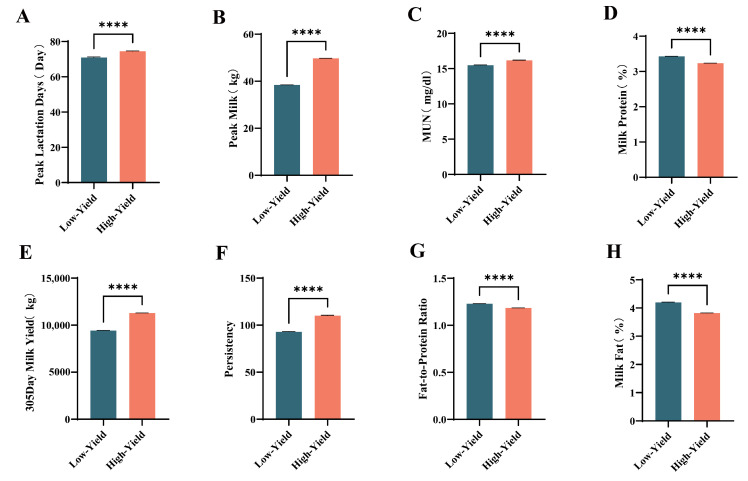
Differences in production performance between high-yield and low-yield dairy cows during (**A**) Peak Lactation Days, (**B**) Peak Milk, (**C**) Milk Urea Nitrogen, (**D**) Milk Protein, (**E**) 305-days Milk Yield, (**F**) Persistency, (**G**) Fat-to-Protein Ratio, (**H**) Milk Fat. Significant differences are indicated as **** *p* < 0.0001.

As illustrated in Figure 1, high-yielding dairy cows exhibited significantly greater peak milk yield days (Figure 1A, *p* < 0.0001), peak milk production (Figure 1B, *p* < 0.0001), milk urea nitrogen levels (Figure 1C, *p* < 0.0001), 305-day milk yield (Figure 1E, *p* < 0.0001), and persistency (Figure 1F, *p* < 0.0001) in comparison to their low-yielding counterparts, with all differences reaching statistical significance. Conversely, the protein concentration (Figure 1D, *p* < 0.0001), fat-to-protein ratio (Figure 1G, *p* < 0.0001), and milk fat concentration (Figure 1H, *p* < 0.0001) in high-yielding cows were found to be significantly lower than those observed in low-yielding cows.

**Figure 2:**
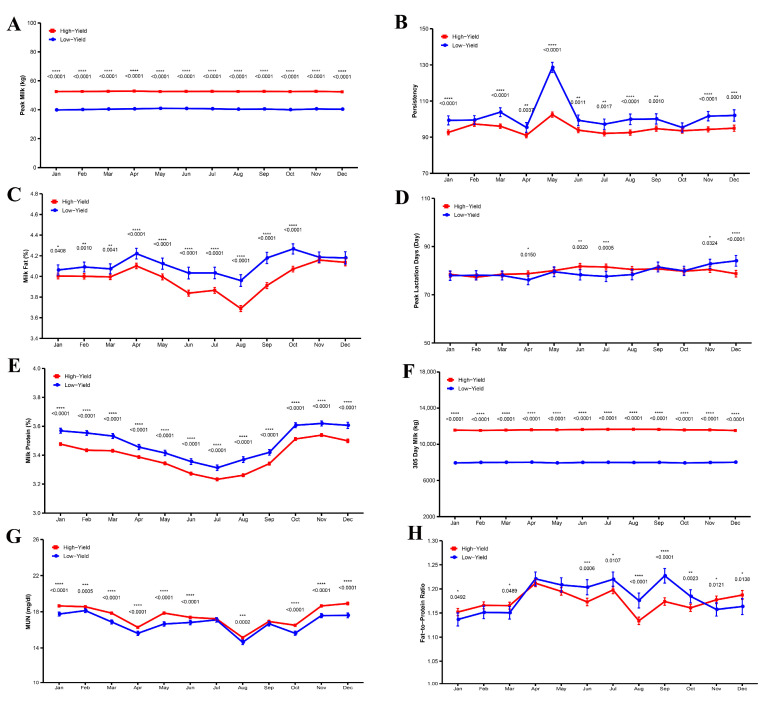
Monthly differences in lactation performance between high and low milk production cows. (**A**) Peak Milk, (**B**) Persistency, (**C**) Milk Fat, (**D**) Peak Lactation Days, (**E**) Milk Protein, (**F**) 305-Day Milk, (**G**) Milk Urea Nitrogen, and (**H**) Fat-to-Protein Ratio. Significant differences are indicated as * *p* < 0.05, ** *p* < 0.01, *** *p* < 0.001, **** *p* < 0.0001.

Figure 2A–H illustrate the impact of seasonal changes on various parameters in high-yield versus low-yield dairy cows. High-yield cows consistently exhibited higher peak milk production and greater persistency throughout all months compared to low-yield cows (*p* < 0.0001). Over the course of the year, high-yield cows showed lower persistency than low-yield cows, with low-yield cows demonstrating notably higher persistency in May, suggesting weaker early lactation performance; this difference was statistically significant (*p* < 0.05). The milk fat percentage in high-yield cows was consistently lower than in low-yield cows year-round, reaching its lowest level in August (*p* < 0.0001). Protein content was also significantly lower in high-yield cows compared to low-yield cows (*p* < 0.0001). Furthermore, the 305-day milk yield of high-yield cows was significantly greater than that of low-yield cows throughout the year (*p* < 0.0001). Regarding urea nitrogen levels, high-yield cows had significantly higher concentrations than low-yield cows (*p* < 0.05), with both groups experiencing declines in April, August, and September. Finally, from April to October, the fat-to-protein ratio in high-yield cows was lower than in low-yield cows, showing a highly significant difference (*p* < 0.0001).

**Figure 3:**
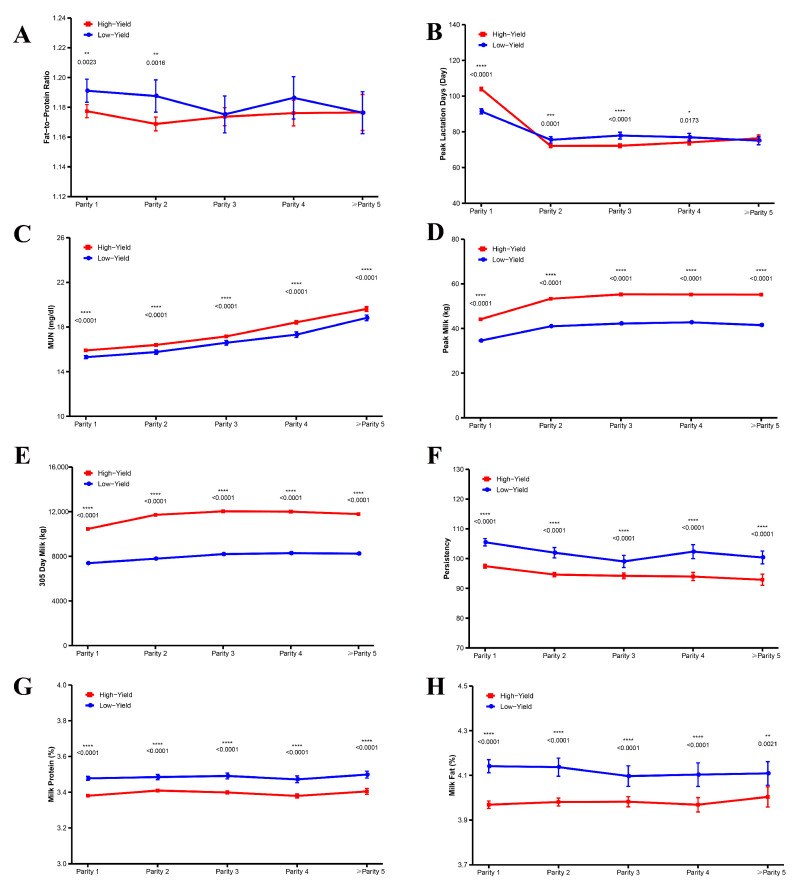
Differences in lactation performance and parity between high and low milk production cows. (**A**) Fat-to-Protein Ratio, (**B**) Peak Lactation Days, (**C**) Milk Urea Nitrogen, (**D**) Peak Milk, (**E**) 305-Day Milk, (**F**) Persistency, (**G**) Milk Protein, and (**H**) Milk Fat. Significant differences are indicated as * *p* < 0.05, ** *p* < 0.01, *** *p* < 0.001, **** *p* < 0.0001.

Figure 3A–H illustrates the influence of lactation number on various parameters in both high-yield and low-yield dairy cows. In low-yield cows, milk fat percentage differed significantly between the first and second lactations (*p* < 0.01). Additionally, after the first lactation, high-yield cows reached their peak milk production in significantly fewer days than low-yield cows (*p* < 0.05). Milk urea nitrogen levels in high-yield cows increased markedly with successive lactations compared to low-yield cows (*p* < 0.0001). Following the first lactation, peak milk yield rose gradually, with high-yield cows exhibiting significantly higher peak yields than low-yield cows (*p* < 0.0001). Moreover, from the second lactation onward, both peak milk yield and 305-day milk production were significantly greater in high-yield cows than in low-yield cows (*p* < 0.0001), reaching their highest levels at the third lactation before declining significantly after the fourth and fifth lactations. Regarding persistency, low-yield cows showed significantly higher values than high-yield cows from the first through fifth lactations (*p* < 0.0001). Furthermore, milk protein and fat percentages were consistently higher in low-yield cows compared to high-yield cows across the first to fifth lactations (*p* < 0.001).

**Figure 4:**
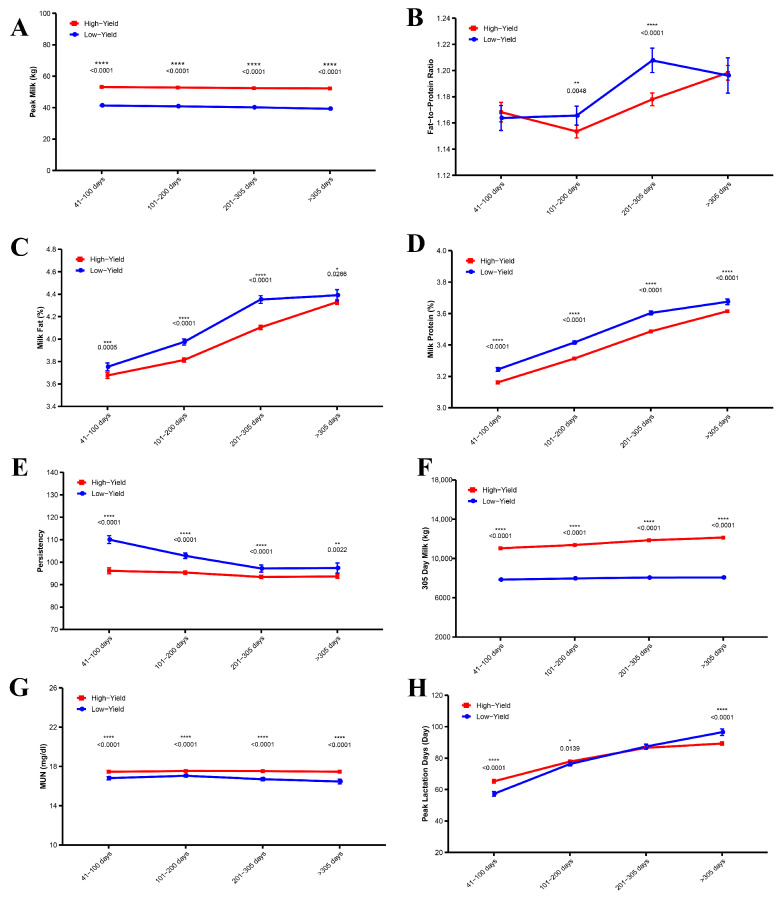
Differences in lactation performance and lactation days between high and low yielding cows. (**A**) Peak Milk, (**B**) Fat-to-Protein Ratio, (**C**) Milk Fat, (**D**) Milk Protein, (**E**) Persistency, (**F**) 305-Day Milk, (**G**) Milk Urea Nitrogen, (**H**) Peak Lactation Days. The significance of the difference is expressed as * *p* < 0.05, ** *p* < 0.01, *** *p* < 0.001, **** *p* < 0.0001.

Figure 4A–H illustrates the effects of lactation duration on various parameters in high-yield and low-yield dairy cows. Over the 0–305 day period, high-yield cows exhibited a significantly higher peak milk yield than low-yield cows, with a highly significant difference (*p* < 0.0001). From days 101 to 305, the fat-to-protein ratio was significantly lower in high-yield cows compared to low-yield cows (*p* < 0.01). Between days 41 and 305, both milk fat percentage and milk protein percentage were significantly lower in high-yield cows than in low-yield cows (*p* < 0.05). Throughout the 0–305 day period, the persistency of high-yield cows was significantly less than that of low-yield cows, with a highly significant difference (*p* < 0.0001); persistency in low-yield cows gradually decreased as lactation progressed. Regarding milk yield at 305 days, high-yield cows consistently produced significantly more milk than low-yield cows throughout the entire lactation period, with a highly significant difference (*p* < 0.0001). Additionally, urea nitrogen levels were significantly higher in high-yield cows than in low-yield cows throughout lactation (*p* < 0.005). Lastly, between days 41 and 100, high-yield cows took longer to reach peak milk production compared to low-yield cows, whereas after day 305, the milk yield of high-yield cows was significantly lower than that of low-yield cows (*p* < 0.0001).

### 3.2. Differences in Somatic Cell Counts Between High- and Low-Yielding Dairy Cows

**Figure 5:**
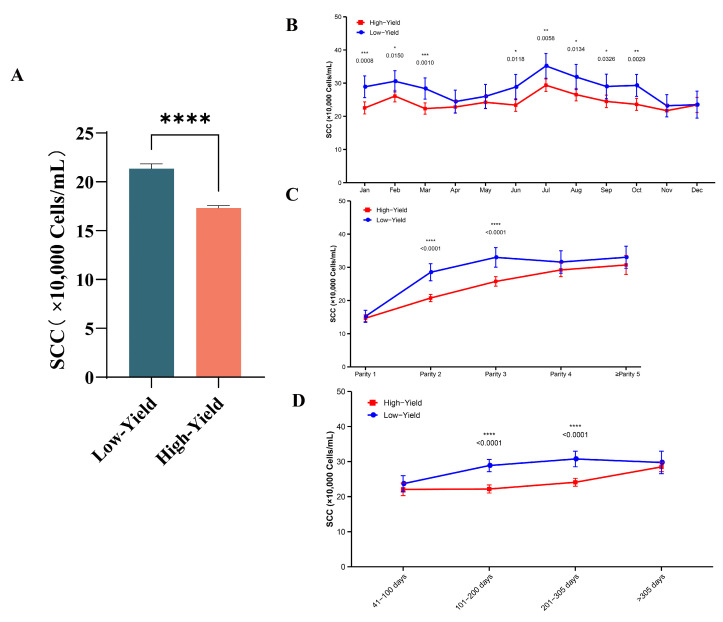
Differences in somatic cells between high and low milk yield cows. (**A**) SCC levels between groups, (**B**) SCC month differences, (**C**) SCC utilization differences in parity, (**D**) SCC differences in lactation days. Significance of differences are indicated as * *p* < 0.05, ** *p* < 0.01, *** *p* < 0.001, **** *p* < 0.0001.

As illustrated in Figure 5A, low-yield dairy cows exhibited a significantly higher SCC during peak lactation compared to high-yield cows (*p* < 0.0001). Monthly analysis revealed that low-yield cows consistently had higher SCC than high-yield cows throughout the year (*p* < 0.05), with noticeable fluctuations influenced by climate. Furthermore, when considering parity, low-yield cows showed significantly elevated SCC over the 2nd to 3rd lactation periods compared to high-yield cows (*p* < 0.0001). Additionally, regarding days in milk, low-yield cows had higher SCC than high-yield cows during the late lactation phase, specifically from day 101 to 305 (*p* < 0.05).

### 3.3. Factors Affecting Milk Yield Based on Multivariate Linear Regression Analysis

In this study, various indicators derived from the DHI data were utilized as independent variables within a linear regression model, with milk yield designated as the dependent variable. The Stepwise selection method was applied to systematically remove non-significant independent variables, leading to the formulation of a regression equation that is both representative and exhibits optimal fit. The resulting equation is expressed as follows: y = −0.012 × parity + 0.011 × season + 0.088 × calving interval − 0.046 × lactation days − 0.131 × milk fat percentage − 0.071 × protein percentage − 0.2549 × fat-to-protein ratio + 0.031 × corrected milk + 0.002 × previous milk yield − 0.004 × previous somatic cell count + 0.006 × previous milk loss − 0.006 × peak milk yield − 0.001 × peak milk Yield days − 0.0003805 × total protein + 1.883.

The number of data entries, mean values, and standard deviations for each variable are presented in Table 2.

**Figure 6:**
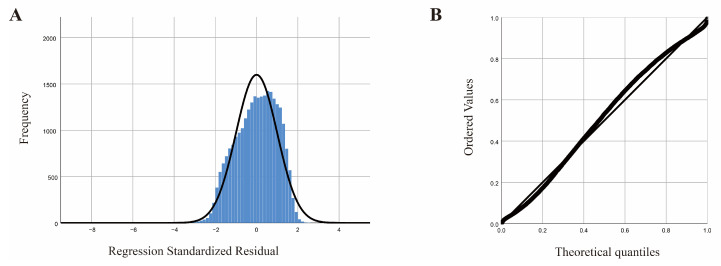
Regression model; (**A**) standardized residual histogram; (**B**) standardized residual normal P-P plot.

The statistical evaluation of the normality test for the residuals indicated that the dataset was appropriately centered, and the residuals from the model exhibited a normal distribution (refer to Figure 6A,B).

The model summary presented in Table 3 reveals that the *R*^2^ value of the linear regression model is 0.665, suggesting that the independent variables account for 66.5% of the variability in milk yield.

As presented in Table 4, the F-test was conducted for the model, yielding an F-value of 2245.954 and a *p*-value of 0.0001, which is less than the significance level of 0.01. This result suggests that at least one independent variable exerts a statistically significant influence on milk yield.

As shown in Table 5, the factors of season, adjusted milk, persistency, total milk fat (%), 305-day milk yield (kg), previous milk loss (kg), and previous milk yield (kg) have a positive impact on milk yield. Meanwhile, the factors of parity (lactation number), days in milk grouping, milk fat percentage (%), milk protein percentage (%), fat-to-protein ratio, mature equivalent (kg), total milk protein (%), total milk yield (kg), peak day (days), peak milk (kg), and somatic cell count have a negative impact on milk yield.

### 3.4. Analysis of Factors Affecting SCC Based on Multiple Linear Regression

In this study, each DHI metric was utilized as an independent variable, while the somatic cell count served as the dependent variable within a linear regression framework. Employing stepwise regression to eliminate non-significant variables, the most representative and optimal fitting equation was derived: y = 0.042 parity − 0.022 season + 0.055 days in milk + 0.602 milk protein − 0.131 milk fat + 0.747 fat-to-protein ratio − 0.026 urea nitrogen 1.163 milk loss − 0.090 milk payment difference − 0.019 milk yield adjustment − 0.003 WHI + 0.219 prior somatic cell count − 0.155 prior milk loss − 0.001 total milk fat + 0.000926 total milk protein. The number, mean, and standard deviation of each variable are in Table 6.

**Figure 7:**
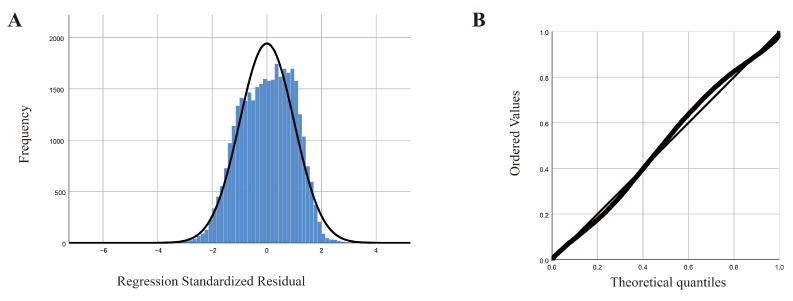
Regression model; (**A**) standardized residual histogram; (**B**) standardized residual normal P-P plot.

The statistical evaluation of the residual normality test indicated a favorable central tendency within the dataset, as the residuals of the model data conformed to a normal distribution (refer to Figure 7A,B).

The model summary presented in Table 7 reveals that the linear regression model exhibits an *R*^2^ value of 0.65. This suggests that the independent variables account for 65% of the variability in the somatic cell count.

As presented in Table 8, the F-test was conducted for the model, yielding an F-value of 2748.199 and a *p*-value of 0.0001, which is less than the significance level of 0.01. This result suggests that at least one of the independent variables exerts a significant influence on the somatic cell count.

As shown in Table 9, the following factors have a positive impact on somatic cell count: parity (lactation number), days in milk, milk protein percentage (%), fat-to-protein ratio, milk loss (kg), prior somatic cell count, peak milk (kg), total milk yield (kg), total milk protein (%), and mature equivalent (kg). On the other hand, the factors of season, milk fat percentage (%), urea nitrogen (mg/dL), milk payment difference, adjusted milk yield, WHI, prior milk loss (kg), 305-day milk yield (kg), and total milk fat (%) have a negative impact on somatic cell count.

### 3.5. Analysis of the Incidence of Clinical Mastitis

**Figure 8:**
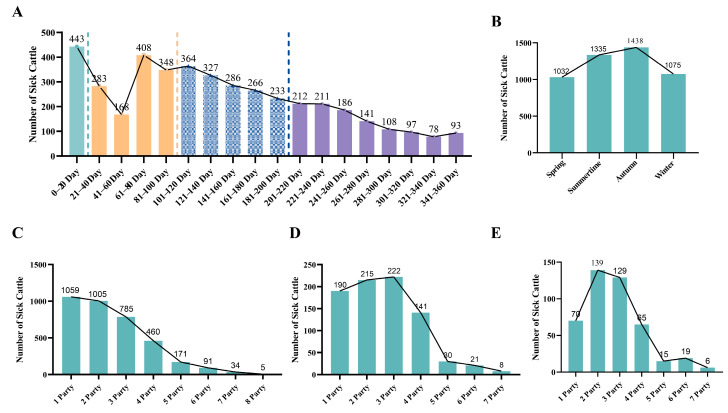
Analysis of the incidence of clinical mastitis in dairy cows. (**A**) Statistics of dairy cows with clinical mastitis during lactation days. (**B**) Statistics of dairy cows with clinical mastitis in different seasons. (**C**) Statistics of the parity of dairy cows with one clinical mastitis during lactation. (**D**) Statistics of the parity of dairy cows with two clinical mastitis during lactation. (**E**) Statistics of the parity of dairy cows with three or more clinical mastitis during lactation.

As illustrated in Figure 8A, the incidence of clinical mastitis among dairy cows initially declines and subsequently rises with an increase in days in milk, ultimately showing a gradual reduction during the mid to late stages of lactation. Figure 8B indicates that the prevalence of clinical mastitis is higher during the summer and autumn months, with recorded cases of 1335 in summer and 1438 in autumn, compared to lower incidences in spring and winter, which report 1032 and 1075 cases, respectively. Furthermore, Figure 8C reveals that the occurrence of a single episode of clinical mastitis is more frequent in cows during their first and second lactations. In contrast, Figure 8D shows that cows experiencing two episodes of clinical mastitis are predominantly found in the second and third lactations. Additionally, Figure 8E highlights that cows with three or more episodes of clinical mastitis are primarily concentrated in the second and third lactations.

## 4. Discussion

Dairy Herd Improvement (DHI) represents a prevalent technological approach in dairy farming. This research involved the collection and analysis of DHI-related data from dairy farms located in the Heilongjiang region of China. The analysis examined various factors, including parity, season, days in milk, and somatic cell count (SCC), with a comparative focus on high-yielding versus low-yielding dairy cows. The results of this study provide significant insights that can inform strategies for enhancing dairy farming efficiency, optimizing management practices, and ensuring the health of dairy cows’ udders [7].

The findings of this study indicate that high-yielding dairy cows exhibit superior production metrics compared to their low-yielding counterparts, specifically in terms of peak milk yield, 305-day milk yield, and lactation persistence. Conversely, high-yielding cows demonstrate significantly lower percentages of milk fat and milk protein, as well as a reduced fat-to-protein ratio in their milk [8]. This observation may be attributed to the “dilution effect,” wherein the increased volume of milk produced by high-yielding cows results in a greater total output of milk fat and protein; however, the concentration of these components is diminished due to the elevated water content in the milk. Furthermore, high-yielding dairy cows exhibit elevated levels of urea nitrogen, suggesting a potential decline in the efficiency of rumen nitrogen metabolism. This may occur as metabolic resources are preferentially allocated towards milk production rather than the maintenance of body tissues, which is consistent with the metabolic adaptation observed in states of negative energy balance [9].

Dairy farming has experienced expansion; however, it remains significantly influenced by the natural environment, particularly in relation to climatic conditions, which exhibit notable seasonality. Cows demonstrate a preference for cooler temperatures over warmer ones, with an optimal growth temperature range of 10–20 °C. Environmental temperatures that fall outside this range, whether excessively low or high, can induce stress responses in cows, leading to a marked decrease in feed intake and, consequently, adversely affecting milk production [10]. The Heilongjiang region is characterized by a temperate and cold temperate continental monsoon climate, which includes spring droughts and low temperatures, hot and rainy summers, autumn floods, early frosts, and prolonged cold winters. The limited frost-free period significantly influences various DHI indicators. Empirical findings indicate that factors such as milk fat percentage, milk protein percentage, peak milk day, urea nitrogen levels, and the fat-to-protein ratio are substantially impacted by seasonal variations. This suggests that the observed results align with the seasonal trends in dairy cow production performance within the Heilongjiang region.

Research indicates that dairy cows achieve their highest milk production during their second lactation, with yields declining in subsequent parities. In this study, both peak milk yield and 305-day milk production were highest between the second and third parities, followed by a decline after the fifth parity. This reduction is likely due to aging and the gradual decline in mammary gland cell function, a pattern observed in both high- and low-producing cows. First-parity cows generally produce less milk because their physiological development is still ongoing. As cows progress through their second, third, and fourth parities, milk production increases and peaks alongside the full maturation of their physiological systems. After the fifth parity, aging and functional deterioration contribute to decreased milk output. Moreover, during peak lactation, high-producing cows exhibit significantly better lactation performance than low-producing cows. Lactation performance also diminishes as the number of days in milk increases, underscoring the importance of this factor in lactation outcomes. While high-producing cows demonstrate greater lactation efficiency at peak production, the cumulative effects of mammary cell fatigue and degeneration over time may eventually reduce their lactation performance.

SCC is a direct indicator of dairy cow udder health, and it is a globally recognized measure of udder health in dairy cows [11,12]. The SCC criteria for mastitis diagnosis in China are as follows: SCC ≤ 2 × 10^5^ cells/mL is considered negative, 2 × 10^5^ cells/mL < SCC ≤ 5 × 10^5^ cells/mL is weakly positive, 5 × 10^5^ cells/mL < SCC ≤ 5 × 10^6^ cells/mL is positive, and SCC > 5 × 10^6^ cells/mL is strongly positive. In this study, the SCC of low-yielding cows was significantly higher than that of high-yielding cows (*p* < 0.0001), and it increased with increasing parity, indicating that udder inflammation accumulates with each lactation. Research has shown that higher SCC values are associated with greater milk loss, poorer milk flavor and quality, decreased milk fat percentage, reduced cheese yield, and shorter shelf-life of liquid milk. Additionally, there is a positive correlation between SCC and the incidence of mastitis. Different SCC ranges correspond to different mastitis incidence rates. High levels of SCC are not only directly related to an increased risk of subclinical mastitis but may also suppress the expression of lactation-related genes and proteins, such as STAT5 and LALBA, through inflammatory factors like IL-6 and TNF-α, leading to decreased milk yield [13].

The impact of season and parity on SCC is also worth paying attention to. In this study, the SCC of low-yielding cows was significantly higher in summer and autumn than that of high-yielding cows. This may be related to the increased risk of udder stress and infection caused by the high temperature and humidity environmental conditions in summer and autumn [14]. In addition, an increase in parity is also associated with elevated SCC. The results indicate that as parity increases, the health status of mammary cells gradually declines, which may be related to the aging and functional degeneration of mammary cells [15]. At the same time, after going through multiple lactation cycles, long-term and frequent milking operations can cause certain mechanical damage to the teats. This damage increases the risk of mastitis infection, which in turn leads to an increase in somatic cell count [16]. At the same time, after going through multiple lactation cycles, long-term and frequent milking operations can cause certain mechanical damage to the teats. This damage increases the risk of mastitis infection, which in turn leads to an increase in somatic cell count.

The incidence of clinical mastitis exhibits a notable pattern, being more prevalent during the mid-to-late stages of lactation and occurring with greater frequency in the summer and autumn months. This trend may be attributed to the heightened risk of udder stress and infection associated with elevated temperature and humidity levels in the environment. Furthermore, it is observed that cows experiencing multiple episodes of clinical mastitis are predominantly in their second or third lactation, suggesting that cows in these parities may possess an increased susceptibility to mastitis.

In the multiple linear regression analysis conducted in this study, the milk yield model (*R*^2^ = 0.665) identifies parity, season, days in lactation, and corrected milk as significant predictors of milk production efficiency. In practical applications, optimizing management strategies during the peak lactation period could prolong the duration of elevated milk yield. The SCC model (*R*^2^ = 0.650) indicates that parity, season, days in lactation, protein percentage, and somatic cell count significantly influence SCC in cow’s milk. This finding underscores the necessity for dairy farms to prioritize health monitoring for cows with high parity and to implement early detection measures for mastitis through regular DHI testing.

The findings of this study offer valuable insights for the dairy industry. However, it is important to note that the data were collected from farms in Heilongjiang Province, China, and the unique climatic and management conditions of this region may limit the generalizability of the conclusions drawn. Future research could benefit from the inclusion of additional variables, such as environmental factors, genetic influences, relevant animal husbandry policies, and fluctuations in the milk market [17,18]. Moreover, it is essential to acknowledge that the mastitis records in this study are confined to clinical cases, potentially leading to an underestimation of the impact of subclinical infections.

## 5. Conclusions

The SCC of high-yielding cows is lower than that of low-yielding cows. The increase in SCC with the rise in parity indicates that the cumulative effect of parity impacts the udder health of cows.

## Figures and Tables

**Table 1:** Data volume statistics.

Ranch	Number of Cows	DHI Date		Bovine Mastitis
		n	Time	n
Ranch A	2055	11,031	From January 2024 to April 2024	4032
Ranch B	534	20,412	From January 2021 to April 2024	223
Ranch C	940	24,389	From January 2021 to November 2023	194
Ranch D	1425	12,489	From January 2023 to April 2024	-
Ranch E	2993	28,847	From January 2023 to November 2023	-
Ranch F	761	39,127	From January 2018 to April 2024	-
Total	8708	136,295		4449

**Table 2:** Descriptive statistics.

	Mean	Standard Deviation	N
Milk Production (kg)	1.78	0.416	26,048
Parity	2.79	1.059	26,048
Season	2.517	1.1751	26,048
Calving Interval (day)	393.726	69.0979	26,048
Lactation Days (day)	3.23	0.702	26,048
Milk Fat (%)	3.89	0.917	26,048
Milk Protein (%)	3.33	0.345	26,048
Fat-to-Protein Ratio (%)	1.17	0.248	26,048
Milk Urea Nitrogen (mg/dL)	16.21	3.606	26,048
Milk Loss	0.64	1.546	26,048
Poor Milk Quality (kg)	1.18	2.896	26,048
Economic Loss	1.851	4.5255	26,048
Energy Corrected Milk (kg)	44.967	11.7882	26,048
Sustaining Force	99.807	51.2047	26,048
WHI	106.060	26.3454	26,048
Previous Milk Yield (kg)	42.894	10.9668	26,048
Previous Somatic Cell Count	2.352	2.0088	26,048
Previous Milk Loss (kg)	0.645	1.5981	26,048
Peak Milk Yield (kg)	53.120	8.9042	26,048
Peak Day (day)	73.53	38.669	26,048
305-Day Milk Yield (day)	11,463.6427	2171.35038	26,048
Total Milk Yield (kg)	8038.2269	3349.11376	26,048
Total Milk Fat (%)	303.4076	131.60021	26,048
Total Milk Protein (%)	258.03	107.919	26,048
Mature Equivalent (kg)	12,057.85	2321.335	26,048

**Table 3:** Model summary.

Model	*R*	*R* ^2^	Adjusted *R*^2^	Standard Error
1	0.815 a	0.665	0.665	0.241

Note: a Explanatory variables: (constant), adult equivalent (kg), milk loss (kg), fat-to-protein ratio, peak day (days), season, calving interval (days), persistence, protein percentage (%), parity (lactation), previous milk loss (kg), milk urea nitrogen (mg/dL), lactation days group, WHI, somatic cell count, previous milk yield (kg), peak milk (kg), total milk fat (kg), corrected milk (kg), total protein (%), total milk yield (kg), milk price difference, milk fat percentage (%), 305-day milk yield (kg). b Dependent variable: milk yield.

**Table 4:** Analysis of variance (ANOVA).

	Sum	Degree of Freedom	Mean Squares	*F*	*p*
Squares Regression	3004.389	23	130.626	2245.954	0.0001 b
Squares Error	1513.567	26,024	0.058		
Total	4517.956	26,047			

Note: a Explanatory variables: (constant), adult equivalent (kg), milk loss (kg), fat-protein ratio, peak day (days), season, calving interval (days), persistence, protein percentage (%), parity (lactation), previous milk loss (kg), milk urea nitrogen (mg/dL), lactation days group, WHI, previous somatic cell count, previous milk yield (kg), peak milk (kg), total milk fat (kg), corrected milk (kg), total protein (%), total milk yield (kg), milk price difference, milk fat percentage (%), 305-day milk yield (kg). b Dependent variable: milk yield.

**Table 5:** Coefficients of multiple linear regression model.

	Unstandardized Coefficients		Standardized Regression Weights	t	*p*	*β* 95%CI	
	β	s					
(Constant)	1.883	0.075		25.052	0.000	1.735	2.030
Parity	−0.012	0.003	−0.030	−4.522	0.000	−0.017	−0.007
Season	0.011	0.001	0.031	8.247	0.000	0.008	0.013
Calving Interval	0.000	0.000	−0.009	−2.373	0.018	0.000	0.000
Lactation Days	−0.046	0.005	−0.077	−9.938	0.000	−0.055	−0.037
Milk Fat	−0.131	0.018	−0.288	−7.420	0.000	−0.165	−0.096
Milk Protein	−0.071	0.022	−0.059	−3.259	0.001	−0.113	−0.028
Fat-to-Protein Ratio	−2.549 × 10^−1^	0.057	−0.152	−4.452	0.000	−0.367	−0.143
Milk Urea Nitrogen	0.000	0.000	0.000	0.073	0.941	−0.001	0.001
Milk Loss	−3.538 × 10^−3^	0.007	−0.013	−0.509	0.611	−0.017	0.010
Poor Milk Quality	0.003	0.004	0.023	0.874	0.382	−0.004	0.011
Economic Loss	0.031	0.000	0.875	89.940	0.000	0.030	0.032
Energy Corrected Milk	0.000	0.000	0.021	4.750	0.000	0.000	0.000
Sustaining Force	0.000	0.000	−0.012	−1.498	0.134	0.000	0.000
WHI	0.002	0.000	0.061	7.749	0.000	0.002	0.003
Previous Milk Yield	−0.004	0.001	−0.018	−2.955	0.003	−0.006	−0.001
Previous Somatic Cell Count	0.006	0.002	0.022	3.691	0.000	0.003	0.009
Previous Milk Loss	−0.006	0.000	−0.136	−18.511	0.000	−0.007	−0.006
Peak Milk Yield	−0.001	0.000	−0.055	−13.906	0.000	−0.001	−0.001
Peak Day	0.000	0.000	0.936	19.927	0.000	0.000	0.000
305-Day Milk Yield	0.000	0.000	−0.478	−23.860	0.000	0.000	0.000
Total Milk Yield	0.000	0.000	0.066	5.462	0.000	0.000	0.000
Total Milk Fat	−3.805 × 10^−4^	0.000	−0.099	−5.287	0.000	−0.001	0.000
Total Milk Protein	0.000	0.000	−0.711	−15.478	0.000	0.000	0.000

**Table 6:** Descriptive statistics.

	Mean	Standard Deviation	N
Somatic Cell Count	2.60	1.991	34,068
Parity	2.83	1.106	34,068
Season	2.505	1.1742	34,068
Calving Interval (day)	392.639	68.4728	34,068
Lactation Days (day)	3.32	0.697	34,068
Milk Fat (%)	3.96	0.931	34,068
Milk Protein (%)	3.37	0.356	34,068
Fat-to-Protein Ratio (%)	1.18	0.247	34,068
Milk Urea Nitrogen (mg/dL)	16.07	3.640	34,068
Milk Loss	0.60	1.451	34,068
Poor Milk Quality (kg)	1.12	2.717	34,068
Economic Loss	1.745	4.2446	34,068
Energy Corrected Milk (kg)	41.860	13.4291	34,068
Sustaining Force	96.220	52.6455	34,068
WHI	99.672	30.0559	34,068
Previous Milk Yield (kg)	40.319	11.9245	34,068
Previous Somatic Cell Count	2.448	2.0046	34,068
Previous Milk Loss (kg)	0.624	1.5260	34,068
Peak Milk Yield (kg)	51.775	9.5294	34,068
Peak Day (day)	73.00	39.327	34,068
305-Day Milk Yield (day)	11,142.4294	2326.53864	34,068
Total Milk Yield (kg)	8142.1513	3364.16667	34,068
Total Milk Fat (%)	308.9372	132.97144	34,068
Total Milk Protein (%)	263.01	108.860	34,068
Mature Equivalent (kg)	11,714.67	2482.893	34,068

**Table 7:** Model summary.

Model	*R*	*R* ^2^	Adjusted *R*^2^	Standard Error
1	0.806 a	0.650	0.650	1.178

Note: a Explanatory variables: (constant), adult equivalent (kg), previous milk loss (kg), fat-to-protein ratio, season, peak day (days), calving interval (days), protein percentage (%), parity (lactation), milk loss (kg), milk urea nitrogen (mg/dL), lactation days, WHI, previous somatic cell count, previous milk yield (kg), peak milk (kg), total milk fat (%), total milk yield (kg), milk price difference, milk fat (kg), 305-day milk yield (kg). b Dependent variable: somatic cell count.

**Table 8:** Analysis of variance (ANOVA).

	Sum	Degree of Freedom	Mean Squares	*F*	*p*
Squares Regression	87,780.873	23	3816.560	2748.199	0.0001 b
Squares Error	47,278.586	34,044	1.389		
Total	135,059.459	34,067			

Note: a Explanatory variables: (constant), adult equivalent (kg), previous milk loss (kg), fat-protein ratio, season, peak day (days), calving interval (days), protein percentage (%), parity (lactation), milk loss (kg), milk urea nitrogen (mg/dL), lactation days, WHI, previous somatic cell count, previous milk yield (kg), peak milk (kg), total milk fat (%), total milk yield (kg), milk price difference, milk fat (kg), 305-day milk yield (kg). b Dependent variable: somatic cell count.

**Table 9:** Coefficients of multiple linear regression model.

	Unstandardized Coefficients		Standardized Regression Weights	t	*p*	*β* 95%CI	
	β	s					
(Constant)	−0.582	0.318		−1.828	0.068	−1.206	0.042
Parity	0.042	0.010	0.024	4.223	0.000	0.023	0.062
Season	−0.022	0.006	−0.013	−3.981	0.000	−0.033	−0.011
Calving Interval	0.000	0.000	0.006	1.941	0.052	0.000	0.000
Lactation Days	0.055	0.020	0.019	2.786	0.005	0.016	0.093
Milk Fat	−0.009	0.074	−0.004	−0.124	0.901	−0.154	0.135
Milk Protein	0.602	0.091	0.108	6.581	0.000	0.423	0.781
Fat-to-Protein Ratio	7.471 × 10^−1^	0.242	0.093	3.088	0.002	0.273	1.221
Milk Urea Nitrogen	−0.026	0.002	−0.047	−13.577	0.000	−0.029	−0.022
Milk Loss	1.163 × 10^0^	0.032	0.847	36.840	0.000	1.101	1.225
Poor Milk Quality	−0.090	0.017	−0.122	−5.304	0.000	−0.123	−0.056
Economic Loss	−0.019	0.001	−0.130	−14.697	0.000	−0.022	−0.017
Energy Corrected Milk	0.000	0.000	−0.008	−1.927	0.054	−0.001	0.000
Sustaining Force	−0.003	0.001	−0.044	−5.766	0.000	−0.004	−0.002
WHI	0.000	0.001	0.000	−0.062	0.950	−0.002	0.002
Previous Milk Yield	0.219	0.005	0.220	41.844	0.000	0.209	0.229
Previous Somatic Cell Count	−0.155	0.007	−0.119	−22.957	0.000	−0.168	−0.142
Previous Milk Loss	0.007	0.001	0.034	4.948	0.000	0.004	0.010
Peak Milk Yield	0.000	0.000	0.008	2.447	0.014	0.000	0.001
Peak Day	0.000	0.000	−0.171	−3.996	0.000	0.000	0.000
305-Day Milk Yield	0.000	0.000	0.154	8.693	0.000	0.000	0.000
Total Milk Yield	−0.001	0.000	−0.062	−5.878	0.000	−0.001	−0.001
Total Milk Fat	9.262 × 10^−4^	0.000	0.051	3.101	0.002	0.000	0.002
Total Milk Protein	0.000	0.000	0.109	2.623	0.009	0.000	0.000

## Data Availability

The original contributions presented in this study are included within the article.

## Abbreviations

DHI: Dairy Herd Improvement. SCC: Somatic Cell Count. MUN: Milk Urea Nitrogen.

## Appendix A

The various indicators in the DHI report are obtained through testing, statistical analysis, and calculation based on the physiological characteristics and biological model data of the tested dairy cows. Proper and accurate analysis of DHI data is key to addressing the cows’ physiological issues as well as management problems on the dairy farm. The DHI testing indicators and their interpretation and application are as follows.

Milk Production (kg): Peak milk yield refers to the highest daily milk production.

Parity: This refers to the number of calvings of the lactating cows being tested, and this indicator can be used to calculate the 305-day milk yield. Generally, healthy herds show higher milk production potential and persistence within the range of 3 to 3.5 lactations.

Calving Interval (day): The interval between calvings in dairy cows.

Lactation Days (day): It refers to the number of days a dairy cow produces milk.

Milk Fat (%): Milk fat percentage refers to the percentage of fat contained in milk and is one of the important indicators for determining the quality of milk.

Milk Protein (%): The milk protein rate is the percentage of protein in milk measured by an infrared analyzer. The level of this indicator is related to the breed, feed, and management practices.

Fat-to-Protein Ratio (%): The fat-to-protein ratio is also a key indicator in the DHI report. It provides valuable guidance for adjusting the concentrate-to-forage ratio in the diet and helps prevent issues such as hoof inflammation, acidosis, and decreased feed intake in dairy cows. This ratio reflects whether the fat and protein levels in the diet are balanced and can be used to monitor the herd for metabolic diseases. The normal range for the fat-to-protein ratio is 1.12 to 1.30 [19].

Milk Urea Nitrogen (mg/dL): The urea nitrogen content in milk can be used to evaluate the utilization level of crude protein in dairy cow diets, nitrogen utilization efficiency, reproductive performance, and more. Constructing optimized and economical diet formulations based on urea nitrogen testing is the most effective way to reduce feeding costs.

Milk Loss: refers to the loss of milk caused by bacterial infection of the udder. It is calculated based on somatic cell count and milk yield. Unit: kg.

Poor Milk Quality (kg): It equals the milk loss multiplied by the current milk price, which is the cost of the lost portion of the milk.

Economic Loss: Total losses caused by mastitis.

Energy Corrected Milk (kg): It is a standard parameter for calculating feeding efficiency. The fat and protein in energy-corrected milk are both adjusted, allowing for a comparison of milk production differences between different breeds and individual cows. Although there are differences between the values of energy-corrected milk and uncorrected milk, both are meaningful. The correction formula for energy-corrected milk is as follows: ECM = (12.82 × kilograms of milk fat) + (7.13 × kilograms of milk protein) + (0.323 × kilograms of milk).

Sustaining Force: It is an indicator reflecting the lactation persistency of individual cows. During the increasing phase of milk production, the persistency is greater than 100, while during the decreasing phase, it is less than 100.

WHI: The Herd Within Index (WHI) is calculated by dividing an individual cow’s adjusted milk yield by the herd’s average adjusted milk yield. WHI is a relative value, with a normal range of 90–110. The WHI value indicates the cow’s relative performance within the herd.

Previous Milk Yield (kg): It refers to the milk yield during the previous lactation period of the cow.

Previous Milk Loss (kg): It refers to the milk loss situation during the previous lactation period.

Peak Milk Yield (kg): It refers to the milk yield on the peak lactation day.

Peak Day (day): Refers to the number of days in milk at which the highest milk yield occurs during the current lactation of a dairy cow, measured in days. For Holstein cows, the peak day typically occurs 60 to 90 days after calving.

305-Day Milk Yield (day): Also known as the 305-day expected milk yield, this indicator represents the projected 305-day milk yield when the lactation period is less than 305 days; when the lactation period reaches or exceeds 305 days, it represents the actual 305-day milk yield. The 305-day milk yield for dairy cows refers to the total milk production from the 5th day after calving up to the 305th day.

Total Milk Yield (kg): Refers to the total milk yield of the cow from the day of calving up to the date of the current measurement. For cows that have completed lactation for a given parity, this represents the milk production for that parity.

Total Milk Fat (%): Refers to the milk fat content of the cow from the day of calving to the date of this measurement.

Total Milk Protein (%): Refers to the milk protein content of the cow from the day of calving to the date of this measurement.

Mature Equivalent (kg): Mature equivalent refers to the 305-day milk yield adjusted to the fifth lactation. Generally, by the fifth lactation, a cow’s body parts have fully developed, and its production performance reaches its peak. Using mature equivalent allows for comparison of the production performance of cows at different lactation numbers throughout the entire lactation period.

Somatic Cell Count: Somatic cell count refers to the number of somatic cells per milliliter of milk. It reflects the health condition of the cow’s udder on the farm and is the most direct indicator of whether a cow has mastitis.
